# Supplementary material for: Fem-1 Gene of Chinese White Pine Beetle (Dendroctonus armandi): Function and Response to Environmental Treatments
Source: Int J Mol Sci. 2024 Sep 26;25(19):10349. doi: 10.3390/ijms251910349 (PMC11477363; doi:10.3390/ijms251910349)
Supplement: Supplementary file 1 [file ijms-25-10349-s001.zip › ijms-3135279-supplementary.pdf]

Supplementary Information

```
1      ggcaaaactcagtgttttgtgagccgcaggattatcaaggtgtttggcgacatgacagca
61     tagtcggcgaagtctacacgcagtcagttcgcattcaaaaaactgtcgttacatatcctg
121    agtATGGATTATAAAAGTGTGGTGTACAATGCTGCTAGAGATGGCAACCTGAATCGGCTA
          M  D  Y  K  S  V  V  Y  N  A  A  R  D  G  N  L  N  R  L     19
181    AAAATCTACTTGCACTGCAACAAAGGAAAAGAGGAGGTGTCAATGCTCGTAGCAGCGAAG
        K  I  Y  L  H  C  N  K  G  K  E  E  V  S  M  L  V  A  A  K     39
241    ACTTCTGGTGCAACACCTTTGGTTATTGCTTGTCGCAATGGACACTACGATGTTGCGGAA
        T  S  G  A  T  P  L  V  I  A  C  R  N  G  H  Y  D  V  A  E     59
301    TACCTGATCGAGCGATGTCATGCCGACATTGAACAGCCGGGATCTGTTATATTTGATGGT
        Y  L  I  E  R  C  H  A  D  I  E  Q  P  G  S  V  I  F  D  G     79
361    GAGACGATCGATGGGGCACCGCCACTCTGGTGCGCTGCAGCCGCTGGACACATGGATATA
        E  T  I  D  G  A  P  P  L  W  C  A  A  A  A  G  H  M  D  I     99
421    GTAAAACTATTGATCAGTCATGGAGCTGAAGTGAATACCATAACTAGAACGAATTCCACT
        V  K  L  L  I  S  H  G  A  E  V  N  T  I  T  R  T  N  S  T     119
481    CCATTGAGGGCCGCCTGCTTTGATGGGCATTTAGAAATAGTGAAATATTTAGTGCAACAC
        P  L  R  A  A  C  F  D  G  H  L  E  I  V  K  Y  L  V  Q  H     139
541    GGTGCAGACATTGAGGTAGCAAATAAGCATGGGCACACGTGCCTAATGATCGCCTGTTAT
        G  A  D  I  E  V  A  N  K  H  G  H  T  C  L  M  I  A  C  Y     159
601    AAAGGCAATATCAAAATAGTGAGATACTTGTTAAGTCTGAAGGCCAGTATTAACCGGAAA
        K  G  N  I  K  I  V  R  Y  L  L  S  L  K  A  S  I  N  R  K     179
661    AGCGTAAAGGGAAATACCGCATTACACGACTGCTCAGAGAGTGGCAGTTTAGAGATTTTA
        S  V  K  G  N  T  A  L  H  D  C  S  E  S  G  S  L  E  I  L     199
721    AAATTGCTCATCGAACATGGAGCTACGATGGACGTGGATTCTTATGGAATGACTCCGCTG
        K  L  L  I  E  H  G  A  T  M  D  V  D  S  Y  G  M  T  P  L     219
781    TTGGCAGCAGCTGTAATGGGTCACAACCACATAGTCGAATATCTTATTAAACTCCCTCAC
        L  A  A  A  V  M  G  H  N  H  I  V  E  Y  L  I  K  L  P  H     239
841    ATTGTTTCTCGAAGAGAACGCATTGATGCGTTGGAGTTGTTAGGGGCCACCTGCGTGGAC
        I  V  S  R  R  E  R  I  D  A  L  E  L  L  G  A  T  C  V  D     259
901    AAGAAGCGGGACATGATCGGAGCCCTGGAATTGTGGAAACGTGCCATGAACGATAGATAC
        K  K  R  D  M  I  G  A  L  E  L  W  K  R  A  M  N  D  R  Y     279
961    AACGGGGATGGATTGCCCATGCCCAAGCCAGCAACACGAAAAGTGGCGGCTTATGATTAC
        N  G  D  G  L  P  M  P  K  P  A  T  R  K  V  A  A  Y  D  Y     299
1021   GTGGTCGAAATTTCGGACACGCGAGCTTTAGATGATCTCATGGCCGATCCAGATGAAATG
        V  V  E  I  S  D  T  R  A  L  D  D  L  M  A  D  P  D  E  M     319
1081   CGAATGCAAGCTTTAGTTATGCGGGAACGAATTTTGGGTCCAGCCCATCCAGACACGAGT
        R  M  Q  A  L  V  M  R  E  R  I  L  G  P  A  H  P  D  T  S     339
1141   TACTACATTCGGTATCGAGGTGCGGTCTATGCAGATGCAGGAAAGTTTAACAGATGTATT
        Y  Y  I  R  Y  R  G  A  V  Y  A  D  A  G  K  F  N  R  C  I     359
1201   GAGCTGTGGAATTATGCTCTCGATATGCAACAAAGCATGCTGGAATGTCTCAGCCCCATG
        E  L  W  N  Y  A  L  D  M  Q  Q  S  M  L  E  C  L  S  P  M     379
1261   ACCCAAAGCAGCTTGTTCAGTTTCACGGAACTCTTTTCGTTCATGATGGGCGAGGAAGGA
        T  Q  S  S  L  F  S  F  T  E  L  F  S  F  M  M  G  E  E  G     399
1321   AAGCACACAAGTCGCGGCCGCCTCGTGCCGCCGGTCGAAGTTTCCGAAATTCTGCGCGTA
        K  H  T  S  R  G  R  L  V  P  P  V  E  V  S  E  I  L  R  V     419
1381   TTTCGAAAAGCCACCAACGAGGTTGAACTGGGCCACCAGATGCTCACTCGACTGCCCAAC
        F  R  K  A  T  N  E  V  E  L  G  H  Q  M  L  T  R  L  P  N     439
1441   CACGATAAGGATATGACGTATTTGACTAGGGTGACGGTGATAACTCTTCATCTGGCGTCG
        H  D  K  D  M  T  Y  L  T  R  V  T  V  I  T  L  H  L  A  S     459
1501   CTCTTGACCAGGATGGCAACTCATGCATCCGTAAGCGCCGAAACTAAGGATCAAATAAAT
        L  L  T  R  M  A  T  H  A  S  V  S  A  E  T  K  D  Q  I  N     479
1561   AAATCCATTTTCTATCTCAACAAACTGGGAATGCGAACCCGGCATGGCCGCACGTTGCTG
```

K S I F Y L N K L G M R T R H G R T L L 499
1621 CATTTGGCGTGCTGCAGAGACGTGGCACTTCTTGTTAGATATCGGGTCTGTGGCTTTCCG
H L A C C R D V A L L V R Y R V C G F P 519
1681 TCGCAGCACCTGATCAAAGTGTTGCTGGAAGTCGGTGCTGATCCCACAGCTCGCGATGAT
S Q H L I K V L L E V G A D P T A R D D 539
1741 GAAGGCAACACACCTCTTCATCTGGCCGCCTTAACCAATCCTTGCACGTCCACAGTAGCC
E G N T P L H L A A L T N P C T S T V A 559
1801 AAATATCTGCTGGATGGGGGAGCTCACTTAGATGAAATTAACAAAAATGGAGAAACCTTT
K Y L L D G G A H L D E I N K N G E T F 579
1861 GCTAGTTTGTTGAAATGCCAACAGGCACACGCTCTTGTTGACGTGATGAAGTACTCTAGT
A S L L K C Q Q A H A L V D V M K Y S S 599
1921 CTCAAATGCGCTGCTGCTAAAGTGATTAAACAGTTCAAAATTCCTTACCGGCGGATTGTC
L K C A A A K V I K Q F K I P Y R R I V 619
1981 CCTCAAGTGTTAGAAGCCTTCCTTGAAGTCCACTGAatgcttgtttgcctggatatttct
P Q V L E A F L E V H * 630
2041 ccattggtactttttgcttgttctatccagatcgggcggaaatgagaaagtgaaactacg
2101 cagcagaatc**aataaa**aaatcaaaaatttcgctttgaattggagtttttaaaaaaaaaaa
2161 aaaaaaaaaaaaaaaaaaa

Figure S1. *fem-1*A sequence of *Dendroctonus armandi*. Lowercase letters indicate untranslated regions (UTRs), while capital letters indicate open reading frames (ORFs). The box represents the start and stop codons, and the bold font indicates a tail signal. The yellow background indicates the position of RNA interference primers.

1 cgaatctcagctgatcgatgtcagaaagctttccagaatccgaattctattttcattttt
61 caatttccacaaacagtcgaaaatagcacggtgttatgattagtacgcatcgattgtgat
121 caaaagaATGAGCGAAGAACGTGAACGTCTCAAATACAAACTCTACTATGCAGCCTGCAC
M S E E R E R L K Y K L Y Y A A C T 18
181 GGGCATGTCGATCTCCTTGTACACCATGTTATCTGAACTAGACAATAACGAGGCAAATCA
G M S I S L Y T M L S E L D N N E A N Q 38
241 ACTCCTAAACGAGAACCTGCCGGACGATGAGGGACAAGAATGCAACCCTCTGGTGGCTGC
L L N E N L P D D E G Q E C N P L V A A 58
301 CGCACGGTTTGGGCATGAAAAAGTGGTTCGGATCCTTCTCAACAAGTTCAATCCCGACAT
A R F G H E K V V R I L L N K F N P D I 78
361 TGAGCAAGAAGGAGTCGTTAAATTCGACGGATACGTTATTGAAGGGGCCACGGCACTTTG
E Q E G V V K F D G Y V I E G A T A L W 98
421 GTGTGCTGCGTGTACCGGGCACCTTAACATAGTGAAAATTTTAGTGACATCCGGGGCTGA
C A A C T G H L N I V K I L V T S G A D 118
481 TGTGAACCATTCAACTAAAACCAATTCCACGCCGTTACGAGCCGCTTGCTTCGATGGTCG
V N H S T K T N S T P L R A A C F D G R 138
541 TCTGGATATAGTCAAGTACCTGACGTGCCATAAGGCCGACATCCATTTGGCAAACAAGTA
L D I V K Y L T C H K A D I H L A N K Y 158
601 CAACAACACTTGCTTGATGATCGCTGCCTATAAAGGGCATGTAGATGTGGTGAGTTTTCT
N N T C L M I A A Y K G H V D V V S F L 178
661 GTTAGAAAACGGAGCCAATCCAAATGAGCGCGCACTATGTGGGGCTACAGCGCTACATTT
L E N G A N P N E R A L C G A T A L H F 198
721 CTCCGCTGAATGTGGACACGTGGATGTAGTCAAGGAACTACTCAGCTACAACGCAATTTT
S A E C G H V D V V K E L L S Y N A I F 218
781 CTACGCAAACGACACAGGCATGACCCCAATCAAATCAGCGGCGGAACGAACGCGGCAGAA
Y A N D T G M T P I K S A A E R T R Q K 238
841 AGTGGTGGCCTATTTAGTAGAGCGGCCAGAGATCAGCAAAGAGGAGCAAGTCGAGGCCTT
V V A Y L V E R P E I S K E E Q V E A L 258
901 GGAGCTGCTGGGGGCTTCTCTAGCCAATGACAAGGAGAACTACAACATCAGCAAAGCCTA

E L L G A S L A N D K E N Y N I S K A Y 278
961 CAAATACCTCCATCAGGCGATGGCGCTTAGGTTTAGTGATCCGGATAGACCGATCAGGAA
K Y L H Q A M A L R F S D P D R P I R K 298
1021 GCCGACAGTTCCCCCGATTCCCGCCTACGAGAACTGGGTGGAAACGCAAACCTTGGCCGA
P T V P P I P A Y E N W V E T Q T L A E 318
1081 ACTAGAAGCCATTGAGGGAAACACGAATAGTCTTCATATGGAGGCCTTGGTTATACGGGA
L E A I E G N T N S L H M E A L V I R E 338
1141 GCGCATTTTGGGTGTTCACAACCATGAACTGCCCCATCCCATCATCTATCGAGGAGCCGT
R I L G V H N H E L P H P I I Y R G A V 358
1201 GTTTGCAGATAATGCCAGATTTGATAGGTGTTTAGAGCTGTGGCTACACGCGTTGAAGCT
F A D N A R F D R C L E L W L H A L K L 378
1261 GCGCCAAAAGAACCACATCCCCGTGGTGAAGGATTTACTCAGATTTGCGCAGGTGTTTTC
R Q K N H I P V V K D L L R F A Q V F S 398
1321 GCAAATGATTCATGTGGGAGTGCAGGTAACTTACGAGCAGGTGATAGAAGTGCTGGCGGC
Q M I H V G V Q V T Y E Q V I E V L A A 418
1381 CGCCATTATAGAGCTCGAACGCAACAAGGAAAAACTGGCGAAGCCCGCGCCGAAAGACGA
A I I E L E R N K E K L A K P A P K D E 438
1441 GCCCGAAACCGTTATGGTAATTTGCTGTTTTATGTACAAAATATTGGATGAAATCGAAAG
P E T V M V I C C F M Y K I L D E I E S 458
1501 CAATCTCACCACCACTCTCTATCTCCTAACAATTCTCACCAAGCTGATGAAGAAGTGCAA
N L T T T L Y L L T I L T K L M K K C N 478
1561 CGAGGAGGAAAAATTCAACGTGCGTCGAATGGTTTTCGCCCTCAATCAGCTGCAGTTAAC
E E E K F N V R R M V F A L N Q L Q L T 498
1621 CTTGAGGAATGGTCAGACGCTGCTGCATCTGGCCTGTAATGCCGAAACGCCCGTGGATGA
L R N G Q T L L H L A C N A E T P V D D 518
1681 TTTCCATACCAATGATATTTGCAAGTTTCCGTGTGCAGAAACGTCGCGATTGCTGATCAG
F H T N D I C K F P C A E T S R L L I R 538
1741 ATGCGGCGCCAACGTCAACGCAATGGACAACGAGCGAAACACTCCTTTGCATGTCATCGT
C G A N V N A M D N E R N T P L H V I V 558
1801 CAATTACCATAAGGCCATTTCCGACTTTCTGACGCTTCATTCGATCATTACGGATCTTAC
N Y H K A I S D F L T L H S I I T D L T 578
1861 GGAAAATGGGGCTCACACGGACATTGTCAATAACAAAGGGGAGACGCCACTGGAATCCTC
E N G A H T D I V N N K G E T P L E S S 598
1921 CACCACAGGTGTAGCTGAGATAATCCTGAAGACTCAAATCAAAATCAGTCTAAAGTGTAT
T T G V A E I I L K T Q I K I S L K C M 618
1981 GGCAGCAAATGCAGTAAAAGTAAACAATATCCCATATCGAGGTTTGGTGCCTTTAGGTCT
A A N A V K V N N I P Y R G L V P L G L 638
2041 GGATGCGTTTATTGAGTTGCATGGCAAAGGGATCGAAAGATGGGAAACGTACAAAACCGA
D A F I E L H G K G I E R W E T Y K T E 658
2101 GCTGAACACCTTATGAttaaacttcaatttagtcatagttcccttactggtggaaagatg
L N T L * 662
2161 caatgtttttttaatattttacagagtttatgattattttttataagtcattgg**aataaa**
2221 gtatcacaataccgctgtgaaatgttaagggttcctattccgattcaatgcaaaaaaaaa
2281 aaaaaaaaaaaaaaaaaaaaaaaaaaaa

Figure S2. *fem-1*B sequence of *Dendroctonus armandi.* Lowercase letters indicate untranslated regions (UTRs), while capital letters indicate open reading frames (ORFs). The box represents the start and stop codons, and the bold font indicates a tail signal. The yellow background indicates the position of RNA interference primers.

1 aaacttttgtgatattggtttttagtcgatttgttcattctcaacatttaagaattgctc

61 gaattaagacagtttttgcccaactATGTTGCACCATATAAAGTCGAATTTCGCCATTGT
M L H H I K S N F A I V 12
121 GAAGAACGAGTTATTTCATGATCTGATGGACGATGTGAGTAGAGCAGGGCCCAGTGCTCG
K N E L F H D L M D D V S R A G P S A R 32
181 ACTCACCTTTGCCATCAGGAACCGATTAGAGGGGTTCTCCTTGATCACTCGAATGGATAT
L T F A I R N R L E G F S L I T R M D I 52
241 TGTGAATCGCAGAAAGTTCAATTGTTCCCCTCTGTTCCTTGCGTGCCAAAAGGGACAACC
V N R R K F N C S P L F L A C Q K G Q P 72
301 CGAAATCGTGGAATATCTAATAACAGTTTGTGGGGCTGACATTGAACTGGAAGGCACCTA
E I V E Y L I T V C G A D I E L E G T Y 92
361 TGAAGTGCTGGACGACCGCTCCGTTCATATCGTTACCCCCTTGTGGTGTGCTGCAGTGTC
E V L D D R S V H I V T P L W C A A V S 112
421 AGGCCATCTGGATGTTCTAAAGATTCTTATGAAGCATCATGCGGACCTAAATGCTGTGTC
G H L D V L K I L M K H H A D L N A V S 132
481 GGATAGTGGATCTACTCCAGTTAGATCAGCCTGTTTTATGACCCATATCGAAGTCGTGAA
D S G S T P V R S A C F M T H I E V V K 152
541 GTTCATGGTAAAAAACGGCGCTGATATTAATAAAGCGAACTATAACGGGGGCACGTGTTT
F M V K N G A D I N K A N Y N G G T C L 172
601 AATCAATTCGGTGCAGAGTCCCAAATTGTGCCTGTTTCTGCTGGAGAATGGGGCCAACGT
I N S V Q S P K L C L F L L E N G A N V 192
661 CAAGGCGAAGGACAACCAGAACAAAACAGCCCTTCACTATGCCATTCAAGAGCACAGGCT
K A K D N Q N K T A L H Y A I Q E H R L 212
721 GGAAACTGCAAAGATGCTAATAGCTTTTGGTGCTGACTGGAACGCGAAATCCAAACATGG
E T A K M L I A F G A D W N A K S K H G 232
781 GGATGACGCCCTACAATTGACGTGTCTGAAAGGGGCTGAAGATATCTTTGAGTATCTGAT
D D A L Q L T C L K G A E D I F E Y L I 252
841 CCAAAATGTGGCGTATCCCCCGGAAAAAATCGCAAACGCTCACGAATTGATGGGCAGCAC
Q N V A Y P P E K I A N A H E L M G S T 272
901 CTTCTTCGACGAACACAACGACGTCTCCTATTGCCGGCACCACTGGAAACGAGCGCTGGA
F F D E H N D V S Y C R H H W K R A L E 292
961 GATTCGAAACCAGCATAATCTACTGCCTAAGAAACCAATGATGACTCCCCTGGCCTGCTA
I R N Q H N L L P K K P M M T P L A C Y 312
1021 CCGTTACGAAAAAGAGTTCGAAACCCCAGAGGAGCTAATGGAAATGGACTTGGACAGGCT
R Y E K E F E T P E E L M E M D L D R L 332
1081 ACGCATACAGAGCCTGCTAATAGTTGAACGGGTCTTGGGGGCCCATCATAAAGACACAAT
R I Q S L L I V E R V L G A H H K D T I 352
1141 ATTTAGGCTGATGTTTCGAGGAGCCGCTTTTGCGGATGTGGTGCGCTATCAGCGCTGTGC
F R L M F R G A A F A D V V R Y Q R C A 372
1201 GGACTTGTGGCGCCGTGCTCTTGAGCTGCGCATTCAGAAAGACACAGTTATTAGTACGGA
D L W R R A L E L R I Q K D T V I S T D 392
1261 TACGTGCTTTTGTGCCCAGGCGCTGGTAAAACACTTGCTGGACTACAACAATCGCTCAGT
T C F C A Q A L V K H L L D Y N N R S V 412
1321 GCTCAACAAAATTGACAATATGGAGAAAAGGTTTGAAGATATCGTCGAGGCTTTTAAGCT
L N K I D N M E K R F E D I V E A F K L 432
1381 ACTGGTGGAGAATAGTGTGGAGGTGCGCCCGCTGCTGCTTATCAGGCCGCAGCACAAAAA
L V E N S V E V R P L L L I R P Q H K K 452
1441 ACAGCTGGATTATTACTCGAAAGTCATTAAGTGCATCACTCATCTAGTTCACCTGATGAT
Q L D Y Y S K V I K C I T H L V H L M I 472
1501 TGAAACTGGAAAATCGGACACAAATAAGGTGATAGTTGAAAAGCTGGTGCGTGAGCTCAT
E T G K S D T N K V I V E K L V R E L I 492
1561 CAAAGACAACATCAAGTGCTTTTTATCCGGTGAGTCTCTGCTGCATTTATGCGTGTCGAA
K D N I K C F L S G E S L L H L C V S K 512
1621 GCTGAACACCGTGAGATCCAGCTATTTCCGAGACGACGATCCAATAATGGTTTTTCCAAG

L N T V R S S Y F R D D D P I M V F P S 532

1681 CATCAGCGTGGTAAAATTGTTGCTAGAGTGTGGCGCCCCCGTTAACGCAAGAAGCGACAA

I S V V K L L L E C G A P V N A R S D N 552

1741 CGGGCTGACGCCGCTACATGTAGCCTCCATCCCCTACAACTACTGCACGGAAATGCTGAG

G L T P L H V A S I P Y N Y C T E M L S 572

1801 CGTATTGCTAAAATTTGGGGCCCATTTGGACCAGCCTGACCGCCGGCACTGCACGGCGTT

V L L K F G A H L D Q P D R R H C T A L 592

1861 GAAGATTTTGCTGAGCAACAAGTACGATTTAGGGAAAGTTTGTCTGCTGGACTACATGAA

K I L L S N K Y D L G K V C L L D Y M N 612

1921 TCTCCGCTGCCTGTGCGCTAGCCAAATTGTGCAAAACAGAATACCATTTATTGGGCAAAT

L R C L C A S Q I V Q N R I P F I G Q I 632

1981 ACCGAAGAGTTTAGAGGCGTTTGTTCACCAACATGATCCAGAGTTCAAAACAACGTAAaa

P K S L E A F V H Q H D P E F K T T * 650

2041 ctcaacaaaacctgtctctaatcagtttagagcgcgtactatttggccgggaggtttgaa

2101 gttataatt**aataaa**gcacaacacgtttaaaaaaaaaaaaaaaaaaaaaaaaaaaaaaaa

2161 aaaaaaaaaaaaa

Figure S3. *fem-1*C sequence of *Dendroctonus armandi*. Lowercase letters indicate untranslated regions (UTRs), while capital letters indicate open reading frames (ORFs). The box represents the start and stop codons, and the bold font indicates a tail signal. The yellow background indicates the position of RNA interference primers.

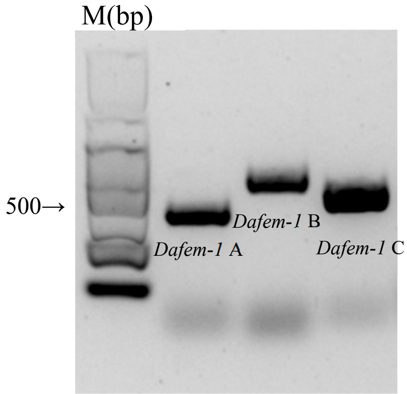

Figure S4. The cDNA amplification of the *Dafem-1* gene was analysed using gel electrophoresis. The DNA size marker is represented by M.

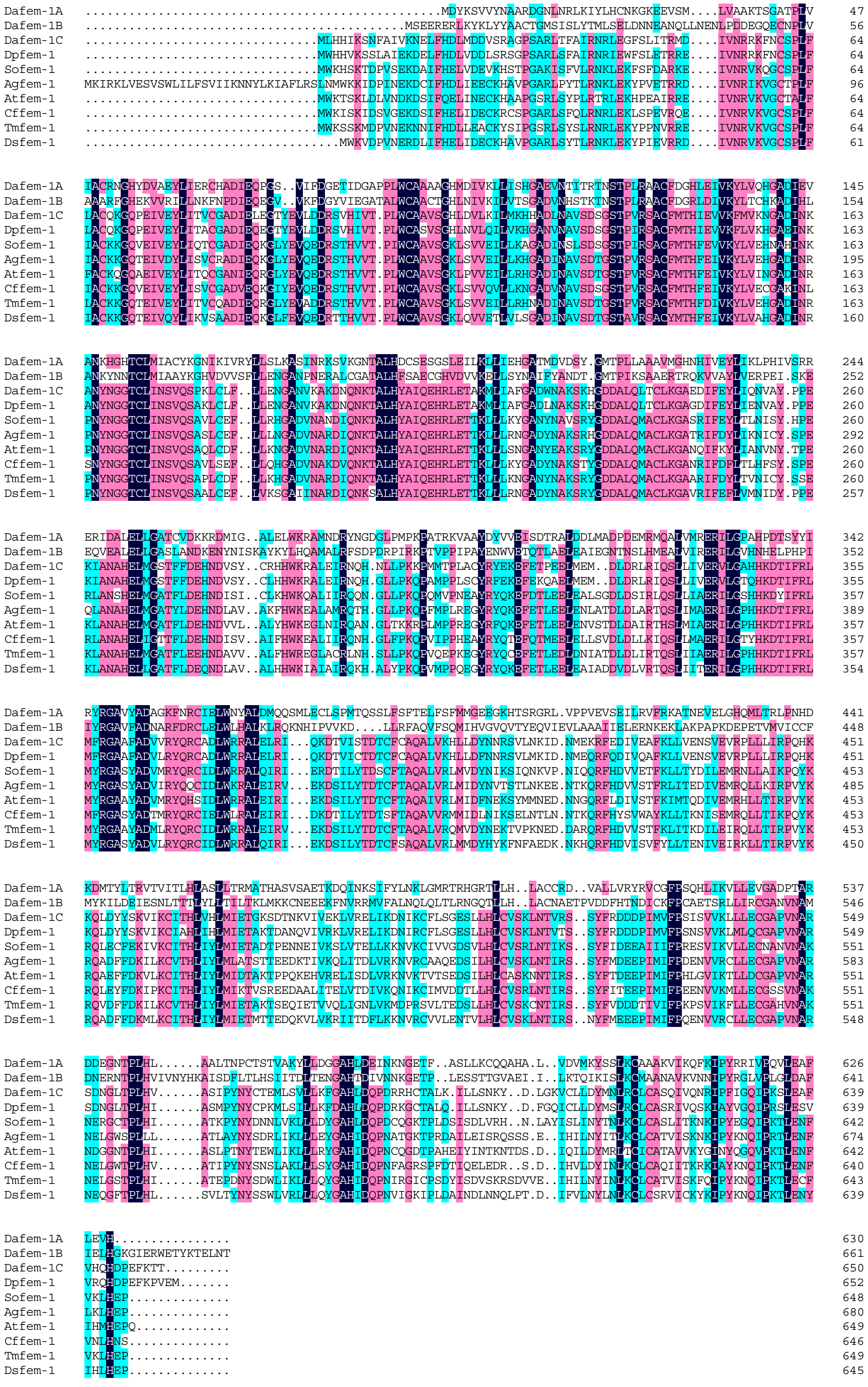

Figure S5. Multiple sequences of the FEM-1 protein from *Dendroctonus armandi*. Other insects were analysed, including *Dendroctonus ponderosae*

(*Dpfem-1*), *Sitophilus oryzae* (*Sofem-1*), *Anoplophora glabripennis* (*Agfem-1*), *Aethina tumida* (*Atfem-1*), *Cylas formicarius* (*Cffem-1*), *Tribolium madens* (*Tmfem-1*) and *Diorhabda sublineata* (*Dsfem-1*). The colours represent the degree of homology, with dark blue indicating 100% homology, pink indicating ≥ 75% homology, and light blue indicating ≥ 50% homology.

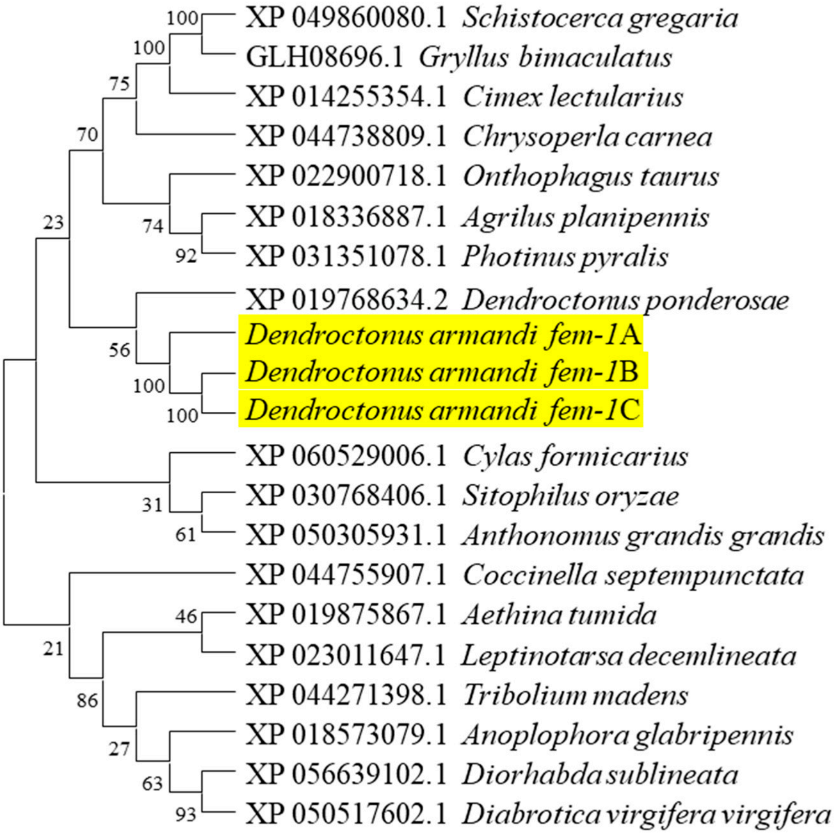

Figure S6. Phylogenetic tree analysis was conducted on the *Dafem-1* gene. The gene was identified by yellow shading, with numbers on the phylogenetic tree branches representing homology. The target gene was also identified by yellow shading, with numbers on the phylogenetic tree branches representing homology. The phylogenetic tree was constructed using the maximum likelihood method with MEGA 11.0. Bootstrap values after 500 pseudo-replicates are shown at nodes. The phylogenetic tree displays bootstrap values (in%) at each branch point, with values below 50% omitted. All species sequences included in the tree belong to Coleoptera.

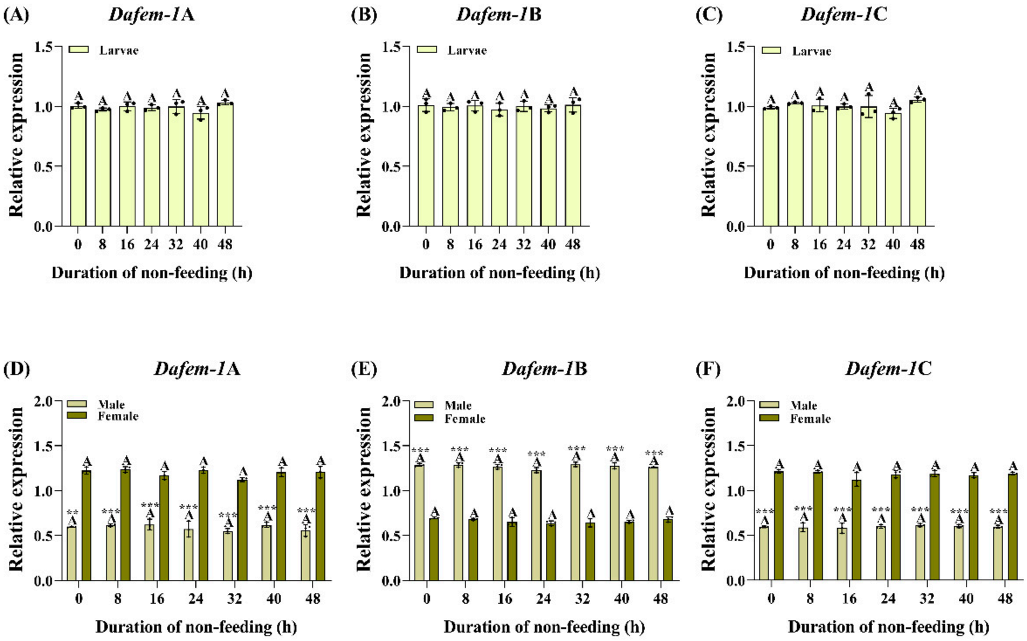

Figure S7. Relative expression of *Dafem-1* genes in non-feeding larvae and adult of *D. armandi*. **(A)** Larvae *Dafem-1*A; **(B)** Larvae *Dafem-1*B; **(C)** Larvae *Dafem-1*C; **(D)** Adult *Dafem-1*A; **(E)** Adult *Dafem-1*B; **(F)** Adult *Dafem-1*C. Relative expression were normalised to *β-actin*. All values are presented as mean ± SE (n=3). Statistical analysis using one-way ANOVA and Tukey's test revealed a significant difference at the $P < 0.05$ level. Capital letters indicate the degree of difference between groups. The asterisk indicates a significant difference between male and female (*$P < 0.05$, **$P < 0.01$, ***$P < 0.001$, independent-sample t-test).

Table S1. Primer sequences used in the research.

Gene Name		Sequence （5′ → 3′）	Purpose
*Dafem-1*A	F	CGCATTACACGACTGCTCAGA	cDNA amplification
	R	AGACCGCACCTCGATACCGAA	
*Dafem-1*B	F	CTGCCCCATCCCATCATCTAT	cDNA amplification
	R	CCAGTGGCGTCTCCCCTTTGT	
*Dafem-1*C	F	GAAACCCCAGAGGAGCTAATG	cDNA amplification
	R	GGATCGTCGTCTCGGAAATAG	
*Dafem-1*A	Inner	GCAGCAGAATCAATAAAAAATCA	3′RACE
	Outer	GGCGGATTGTCCCTCAAGTGTTA	
*Dafem-1*B	Inner	GTCTGGATGCGTTTATTGAGTTGC	3′RACE
	Outer	ATCCTGAAGACTCAAATCAAAATC	
*Dafem-1*C	Inner	CGATTTAGGGAAAGTTTGTCTGC	3′RACE
	Outer	GCTCATCAAAGACAACATCAAGT	
*Dafem-1*A	Inner	ATCCTGCGGCTCACAAAACACTG	5′RACE
	Outer	CAAGTAGATTTTTAGCCGATTCA	
*Dafem-1*B	Inner	AGTAGAGTTTGTATTTGAGAC	5′RACE
	Outer	AACCGTGCGGCAGCCACCAGA	
*Dafem-1*C	Inner	GGCACGCAAGGAACAGAGGGGAA	5′RACE
	Outer	GCACACCACAAGGGGGTAACGAT	
*Dafem-1*A	F	ATATCGGGTCTGTGGCTTTC	RT-qPCR
	R	AGATATTTGGCTACTGTGGACG	
*Dafem-1*B	F	AAGCCTACAAATACCTCCATCAG	RT-qPCR
	R	GTTTCCACCCAGTTCTCGTAG	
*Dafem-1*C	F	GACAACCAGAACAAAACAGCC	RT-qPCR
	R	TTCAGCCCCTTTCAGACAC	
β-actin	F	CATCAGGAAGGACTTGTA	RT-qPCR
	R	GATTCGTCGTATTCTTGTT	
*Dafem-1*A	F	TAATACGACTCACTATAGGGGGGAAATACCGCATTACACGACT	dsRNA synthesis
	R	TAATACGACTCACTATAGGGGACTTGTGTGCTTTCCTTCCTCG	
*Dafem-1*B	F	TAATACGACTCACTATAGGGTCCCGACATTGAGCAAGAAG	dsRNA synthesis
	R	TAATACGACTCACTATAGGGCTAGAGAAGCCCCCAGCAGC	
*Dafem-1*C	F	TAATACGACTCACTATAGGGCGTTACCCCCTTGTGGTGTGC	dsRNA synthesis
	R	TAATACGACTCACTATAGGGTCGTGAGCGTTTGCGATTTTT	

Note: The primer sequence of the connector has been underlined.

Table S2. Percentage identity of the dsRNA nucleotide sequence of *Dafem-1*A, *Dafem-1*B and *Dafem-1*C.

Gene Name	*Dafem-1*A	*Dafem-1*B	*Dafem-1*C
*Dafem-1*A	-	42.91%	39.96%
*Dafem-1*B	42.91%	-	45.62%
*Dafem-1*C	39.96%	45.62%	-
